# Fixation of flail chest or multiple rib fractures: current evidence and how to proceed. A systematic review and meta-analysis

**DOI:** 10.1007/s00068-018-1020-x

**Published:** 2018-10-01

**Authors:** Reinier B. Beks, Jesse Peek, Mirjam B. de Jong, Karlijn J. P. Wessem, Cumhur F. Öner, Falco Hietbrink, Luke P. H. Leenen, Rolf H. H. Groenwold, Roderick M. Houwert

**Affiliations:** 1 Department of Surgery, University Medical Center Utrecht, PO Box 85500, 3508 GA Utrecht, The Netherlands; 2 Utrecht Traumacenter, Utrecht, The Netherlands; 3 Julius Center for Health Sciences and Primary Care, UMC Utrecht, Utrecht, The Netherlands; 4 Department of Clinical Epidemiology, Leiden University Medical Center, Leiden, The Netherlands

**Keywords:** Flail chest, Multiple rib fractures, Operative treatment, Nonoperative treatment, Current evidence

## Abstract

**Purpose:**

The aim of this systematic review and meta-analysis was to present current evidence on rib fixation and to compare effect estimates obtained from randomized controlled trials (RCTs) and observational studies.

**Methods:**

MEDLINE, Embase, CENTRAL, and CINAHL were searched on June 16th 2017 for both RCTs and observational studies comparing rib fixation versus nonoperative treatment. The MINORS criteria were used to assess study quality. Where possible, data were pooled using random effects meta-analysis. The primary outcome measure was mortality. Secondary outcome measures were hospital length of stay (HLOS), intensive care unit length of stay (ILOS), duration of mechanical ventilation (DMV), pneumonia, and tracheostomy.

**Results:**

Thirty-three studies were included resulting in 5874 patients with flail chest or multiple rib fractures: 1255 received rib fixation and 4619 nonoperative treatment. Rib fixation for flail chest reduced mortality compared to nonoperative treatment with a risk ratio of 0.41 (95% CI 0.27, 0.61, *p* < 0.001, *I*^2^ = 0%). Furthermore, rib fixation resulted in a shorter ILOS, DMV, lower pneumonia rate, and need for tracheostomy. Results from recent studies showed lower mortality and shorter DMV after rib fixation, but there were no significant differences for the other outcome measures. There was insufficient data to perform meta-analyses on rib fixation for multiple rib fractures. Pooled results from RCTs and observational studies were similar for all outcome measures, although results from RCTs showed a larger treatment effect for HLOS, ILOS, and DMV compared to observational studies.

**Conclusions:**

Rib fixation for flail chest improves short-term outcome, although the indication and patient subgroup who would benefit most remain unclear. There is insufficient data regarding treatment for multiple rib fractures. Observational studies show similar results compared with RCTs.

**Electronic supplementary material:**

The online version of this article (10.1007/s00068-018-1020-x) contains supplementary material, which is available to authorized users.

## Introduction

Rib fractures are very common in patients with thoracic trauma and nowadays still associated with significant morbidity and mortality due to the underlying injuries to the lung and heart resulting in more pulmonary complications [1–4]. Compared to multiple rib fractures, flail chest is associated with a worse outcome due to a higher incidence of respiratory compromise and concomitant injuries [5, 6].

A combination of adequate pain control, respiratory assistance, and physiotherapy is considered the gold standard in management of rib fractures [3]. Over the past decades, there has been a growing interest in rib fixation for flail chest and for multiple rib fractures, however, there is no consensus regarding the indication and patient selection for rib fixation.

In the field of (orthopedic) trauma surgery, there is increasing scientific evidence that inclusion of observational studies could add value to meta-analyses without decreasing quality of the results [7–10]. Adding observational studies result in larger sample sizes and might enable the evaluation of small treatment effects, subgroups, and infrequent outcome measures while also providing information about the generalizability of the results [11].

The aim of this systematic review and meta-analysis was (1) to present current evidence on outcome after rib fixation compared to nonoperative treatment for both flail chest and multiple rib fractures and (2) to compare effect estimates obtained from RCTs and observational studies.

## Methods

This review was performed according to the Preferred Reporting Items for Systematic Reviews and Meta-Analyses (PRISMA) and the Meta-analysis of Observational Studies in Epidemiology (MOOSE) guidelines [12, 13]. A published protocol for this review does not exist. Ethical committee approval did not apply to this study.

### Search strategy and eligibility criteria

A structured literature search was conducted in MEDLINE, Embase, CENTRAL and CINAHL on June 16th, 2017 for both randomized controlled trials (RCTs) and observational studies comparing operative to nonoperative treatment of traumatic rib fractures. The search was not restricted by publication date, language, or other limits. The full search syntax is provided in Appendix 1.

All obtained studies from the literature search were independently screened for eligibility based on title and abstract by two reviewers (RBB, JP). Exclusion criteria were animal studies, abstracts of conferences, case-reports, reviews, inclusion of patients younger than 18 years, and studies written in another language than English, French, Dutch or German. Disagreement regarding study selection was resolved by discussion with a third reviewer (RMH). References of included studies were manually screened and citation tracking was conducted using Web of Science to identify additional relevant studies.

### Data extraction

Data were extracted by two independent reviewers (RBB, JP), using a data extraction file. Extracted data included first author, year of publication, study period, study design, country, fracture type, number of fractured ribs, number of included patients, number of patients with flail chest or multiple rib fractures (according to the definition used by the original study), age, gender, type of operative treatment, type of nonoperative treatment, duration of follow-up, loss to follow-up, Injury Severity Score (ISS), Abbreviated Injury Scale (AIS), Glasgow Coma Scale (GCS), hemothorax, pneumothorax, pulmonary contusion, type of implant in operative group, mortality during hospitalization, hospital length of stay (HLOS), intensive care unit length of stay (ILOS), duration of mechanical ventilation (DMV), incidence of pneumonia, need for tracheostomy, complications, revision surgery, and implant removal.

### Outcome measures

The primary outcome measure was mortality during hospitalization. Secondary outcome measures were HLOS, ILOS, DMV, incidence of pneumonia, need for tracheostomy, complications, revision surgery, and implant removal.

### Quality assessment

The Methodological Index for Non-Randomized Studies (MINORS) score was used to assess the included studies [14]. The MINORS is a critical appraisal instrument developed to assess the methodological quality of observational surgical studies. Other quality assessment tools focus on a specific study design while the MINORS is externally validated on RCTs and is therefore a suitable instrument for meta-analyses of different study designs. The MINORS score ranges from 0 to 24 and a higher score reflects better quality. Studies were independently assessed by two reviewers (RBB, JP) using the MINORS criteria and disagreement was resolved by discussion with a third reviewer (RMH). Additional details on the MINORS criteria and scoring system are set out in Appendix 2.

### Statistical analysis

Statistical analyses were performed using Review Manager (RevMan, Version 5.3.5 Copenhagen: The Nordic Cochrane Centre, The Cochrane Collaboration, 2014). Data were converted to a mean with standard deviation (SD) using different methods as described in the Cochrane Handbook for Systematic Reviews of Interventions [15].

Different studies based on the same patient cohort were included only once in the analysis [16, 17]. Studies reporting on specific patient subgroups were split and included separately for meta-analysis, provided sufficient information was reported; Qiu et al. distinguished between the presence or absence of a flail chest and Voggenreiter et al. made subgroups based on the presence or absence of pulmonary contusion [18, 19]. Results from both RCTs and observational studies were pooled in the primary analysis.

Meta-analysis was performed if outcome measures of two or more studies were available. For continuous outcome measures, the inverse variance weighted random effects model was used to estimate the pooled difference in the outcome measure for fixation versus no fixation, with corresponding 95% confidence interval (CI). For dichotomous outcomes, we applied the Mantel–Haenszel method and pooled results are presented as risk ratios (RR) with 95% CI. Heterogeneity between studies was assessed by visual inspection of the forest plots and by estimating statistical measure for heterogeneity, i.e., the *I*^2^ statistic. Inspection of a funnel plot of the study-specific difference in the primary outcome measure against its standard error was done to detect potential publication bias. A two-sided *p* value < 0.05 was considered statistically significant.

### Subgroup and sensitivity analyses

In subgroup analysis, we stratified by study design and pooled effects of RCTs were compared with pooled effects of observational studies. For the analysis of study quality only studies with an arbitrarily chosen MINORS score of 16 or higher were included, similar to previously published meta-analyses in orthopedic trauma surgery studying both study designs [8, 10, 20]. To assess the impact of improvement in intensive care management over time, we performed a sensitivity analysis including only studies published in the last 5 years. Different methods were used to include studies with zero events in one or both arms of the outcome measure. To assess the sensitivity of the analyses to the choice of the method of analysis, also the crude methods, DerSimonian–Laird method with correction, the inverse variance with and without correction for zero event data, and the Peto method were applied and results were compared for consistency [21].

## Results

### Search

**Fig. 1:**
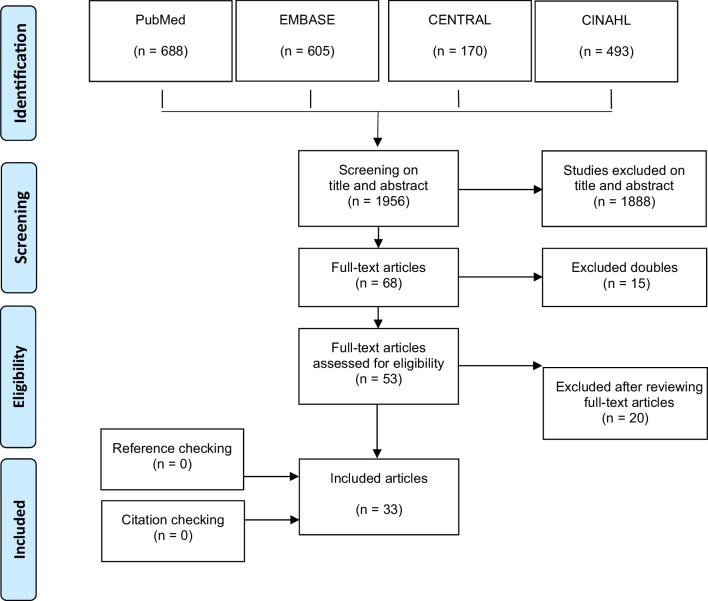
Flowchart of the literature search

The flowchart of the literature search is presented in Fig. 1. Ultimately, 33 studies were included [16–19, 22–50]. There were three RCTs, two prospective cohort studies, 14 retrospective cohort studies, and 14 case–control studies.

### Patient characteristics

The studies included for meta-analysis included 5874 patients; 1255 received rib fixation and 4619 received nonoperative treatment. In the majority of the studies (*n* = 20), patients were surgically treated with plates (Tables 1, 2). Other surgical methods were K-wires and Judet or Adkins struts. Nonoperative treatment consisted generally of ‘best medical treatment’ and included adequate pain management, lung physiotherapy and respiratory support. The weighted average age was 52.9 years and 73% of patients were male. The weighted average of the number of rib fractures was 6.9 in the rib fixation group and 6.0 in the nonoperative group with a weighted mean ISS of 21.2 and 22.4, respectively.

**Table 1:** Baseline characteristics of the included studies comparing rib fixation versus nonoperative treatment of traumatic rib fractures

Study	Study design	Country	Number of patients		Follow-up (months)	Age (years, range or ± SD)	Male (%)	Number of fractured ribs	ISS score
Dehghan et al. (2018) [43]	RC	Canada	RF	77	NR	52 ± 18	55 (76)	NR	NR
			NOM	1631		58 ± 18	1176 (72)		
Ali-Osman et al. (2018) [42]	RC	USA	RF	64	NR	68.5 [63–74]	41 (64)	7 [5.25-9]	17.5 [9–25}
			NOM	135		72 [66–81]	73 (54)	5 [3-7.25]	14 [8–24]
Wijffels et al. (2018) [41]	CC		RF	20	NR	60 [41–69]	15 (75)	9 [8–11]	31 [21–48]
			NOM	20		57 [44–69]	15 (75)	10 [9–14]	32 [21–41]
Kane et al. (2018) [44]	RC		RF	116	NR	58.3 ± 14.4	NR	NR	21.6 (9.1)
			NOM	1000		46.9 ± 29.3			16.1 (11.4)
Fitzgerald et al. (2017) [33]	CC	USA	RF	23	NR	68 (63–89)	NR	NR	21 (16–26)
			NOM	50		75 (65–97)			19 (14–23)
Farquhar et al. (2016) [39]	CC	Canada	RF	19	21.9 ± 13.2	53 ± 14	15 (79)	NR	31.4 ± 9.6
			NOM	36	16.0 ± 12.1	57 ± 16	25 (69)		29.3 ± 8.1
Pieracci et al. (2016) [37]	PC	USA	NOM	35	16.0 [10.0, 23.0]	50 + 15	24 (69)	9.0 [6.0, 13.0]	22.0 [17.0,38.0]
Defreest et al. (2016) [38]	RC	USA	RF	41	28.3 (9–69)	51 (19–80)	32 (78)	11.2 (6–19)	27.5 (16–48)
			NOM	45	13.0 (3–43)	56 (23–89)	39 (87)	10.6 (6–23)	29.3 (16–66)
Uchida et al. (2016) [30]	CC	Japan	RF	10	NR	63 [51, 72]	7 (70)	5 [4, 8]	NR
			NOM	10		57 [53, 75]	7 (70)	5 [2, 7]	
Velasquez et al. (2016) [47]	CC	USA	RF	20	6 [4, 10]	51 [41, 63]	NR	5 [4, 8]	9 [9, 16]
			NOM	20	16 [11, 22]	45 [36, 55]		5 [4.6, 5]	13 [9, 17]
Qiu et al. (2016a) [18]	RC	China	RF	21	NR	35 ± 13	15 (48)	6.0 ± 1.3	NR
			NOM	17		36 ± 14	12 (71)	5.9 ± 1.3	
Qiu [18]	RC	China	RF	65	NR	38 ± 12	46 (71)	3.2 ± 1.2	NR
			NOM	59		36 ± 12	42 (71)	3.5 ± 1.2	
Jayle et al. (2015) [51]	CC	France	RF	10	21.7 ± 7.8	48 ± 11	8 (80)	7.7 ± 2.4	21.7 ± 7.80
			NOM	10	32.3 ± 19.3	51 ± 13	8 (80)	6.6 ± 2.9	32.3 ± 19.3
Zhang Y (2015) [25]	RC	China	RF	24	38 33, 54.25]	43 [34, 50]	19 (79)	11.5 [8, 15.3]	38 [34, 43]
			NOM	15	60 [38, 99.75]	47 [35, 55]	14 (93)	11 [7, 16]	38 [35, 43]
Zhang X (2015) [46]	CC	China	RF	23	419,4 ± 107.1	58 ± 12	21 (72)	7.8 ± 1.5	NR
			NOM	29	419,4 ± 107.1	60 ± 10	16 (70)	7.4 ± 1.7	
Wada et al. (2015) [34]	CC	Japan	RF	84	33 (24–45)	NR	59 (70)	NR	NR
			NOM	336	42 (23–58)		225 (76)		
Wu et al. (2015) [32]	PC	China	RF	75	15.3 ± 6.4	52 ± 5	75 (100)	8.1 (6–12)	NR
			NOM	89	26.5 ± 6.9	51 ± 3	89 (100)	7.9 (6–11)	
Majercik et al. (2015) [16]	CC	USA	RF	137	11.4 + 5.7	56 ± 16	110 (80)	6.5 ± 2.0	21 ± 10.7
			NOM	274	12.3 + 9.1	55 ± 20	56 (80)	4.6 ± 2.3	22 ± 11.8
Xu et al. (2015) [28]	RC	China	RF	17	NR	36 ± 14	12 (71)	6.8 ± 2.1	21.8 ± 7.8
			NOM	15		39 ± 12	12 (80)	7.4 ± 1.6	24.0 ± 8.0
Granhed and Pazooki (2014) [43]	CC	Sweden	RF	60	NR	NR	53 (77)	7.5 (2–14)	21.7 ± 10.7
			NOM	153		NR	NR	NR	30.9 ± 13.3
Doben et al. (2014) [40]	CC	USA	RF	10	21.6 (8–59)	47 ± 15	9 (90)	8.3 (4–20)	26.3 ± 9.5
			NOM	11	28.5 (6–50)	57 ± 17	7 (64)	9.2 (6–16)	35.7 ± 12.7
Marasco et al. (2013) [50]	RCT	Australia	RF	23	90	58 ± 17	20 (87)	11.0 ± 3.1	35.0 ± 11.4
			NOM	23	90	59 ± 10	20 (87)	11.3 ± 4.7	30.0 ± 6.3
Khandelwal et al. (2011) [29]	PC	India	RF	31	30	47	40 (66) = total group	3.1	NR
			NOM	29	30	45		3.3	
Moya et al. (2011) [31]	CC	USA	RF	16	18 ± 12	45 ± 16	14 (88)	8 ± 4	24 ± 7
			NOM	32	16 ± 11	47 ± 14	26 (81)	8 ± 3	25 ± 9
Althausen et al. (2011) [26]	CC	USA	RF	22	17.84 ± 4.51	48	17 (74)	5.9	25.1
			NOM	28	NR	51	23 (79)	7.3	24.3
Solberg et al. (2009) [24]	RC	USA	RF	9	16.1 ± 6.7	39 ± 17	6 (67)	NR	24.9 ± 6.5
			NOM	7	12.0 ± 2.3	41 ± 13	5 (71)		24.8 ± 6.2
Nirula et al. (2006) [35]	CC	USA	RF	30	NR	52	NR	NR	25.7
			NOM	30		50			27.5
Granetzny [23]	RCT	Germany	RF	20	2	41 ± 8	17 (85)	4.4	16.8 ± 3.5
			NOM	20	2	36 ± 15	16 (80)	4.0	18.0 ± 5.1
Balci et al. (2004) [45]	RC	Turkey	RF	27	NR	35 ± 8	20 (74)	NR	21.0 ± 7.4
			NOM	37		31 ± 10	28 (76)		18.4 ± 8.1
Tanaka et al. (2002) [22]	RCT	Japan	RF	18	360	43 ± 12	12 (67)	8,2 ± 3.3	33 ± 11
			NOM	19	360	46 ± 9	14 (74)	8.2 ± 2.6	30 ± 8
Voggenreiter (1996a) [19]	RC	Germany	RF	10	NR	55 ± 8	NR	NR	31.0 ± 7.0
			NOM	18		44 ± 19			36.6 ± 12.3
Voggenreiter (1996a) [19]	RC	Germany	RF	10	NR	50 ± 16	NR	NR	37.0 ± 7.9
			NOM	4		48 ± 27			37.8 ± 19.5
Ahmed and Mohyuddin (1995) [37]	RC	United Arab Emirates	RF	26	(3–9)	20–60 (range)	23 (88)	NR	NR
			NOM	38	(3–9)	10–60 (range)	36 (95)		NR
Kim et al. (1981) [49]	RC	France	RF	18	NR	NR	NR	NR	NR
			NOM	142					
Aubert et al. (1981) [48]	RC	France	NOM	224	NR	NR	NR	NR	NR

*CC* case control, *PC* prospective cohort, *RC* retrospective cohort, *RCT* randomized controlled trial, *RF* rib fixation, *NOM* nonoperative treatment, *NR* not reported

**Table 2:** Treatment characteristics of the included studies comparing operative versus nonoperative management of traumatic rib fractures

Study	Treatment groups	Included fractures	Flail chest in surgery group *n* (%)	Indication for surgery
Dehghan et al. (2018) [43]	NR	FC	77 (100%)	NR
Ali-Osman et al. (2018) [42]	RF: plates + screws	FC + MRF	NR	Displaced rib fractures, uncontrolled pain, rib crepitus with breathing
	NOM: aggressive pain management			
Wijffels et al. (2018) [41]	RF: plates + intramedullary nails	FC	20 (100%)	Flail chest
	NOM: supportive management			
Kane et al. (2018) [44]	RF: NR	FC + MRF	75 (65%)	3 consecutively displaced rib fractures plus FEV1 and FVC less than 50% predicted
	NOM: aggressive multimodal analgesia protocol			
Fitzgerald et al. (2017) [33]	RF: plates + screws	FC + MRF	NR	NR
	NOM: NR			
Farquhar et al. (2016) [39]	RF: plates + screws	FC	19 (100%)	FC (≥ 3 fractures), displaced, segmental rib fractures with respiratory insufficiency
	NOM: standard conservative treatment			
Pieracci et al. (2016) [37]	RF: titanium plates + screws	FC + MRF	28 (80%)	FC (≥ 3 fractures), ≥ 3 displaced fractures; ≥ 30% thorax volume loss, failure treatment within first 72 h
	NOM: standard conservative treatment			
Defreest et al. (2016) [38]	RF: titanium locking plates + screws	FC	41 (100%)	Failure to wean, intractable pain, or respiratory failure
	NOM: NR			
Uchida et al. (2016) [30]	RF: titanium plates + locking screws	FC + MRF	NR	Flail segment, massive dislocation, > 15 mm fracture overlapping, or pain
	NOM: conservative management + chest strap			
Velasquez et al. (2016) [47]	RF: Thoracic Osteosynthesis System (STRATOS)	FC + MRF	NR	FC (≥ 3), ≥ 3 ribs fractured + respiratory failure, intractable pain, thorax deformity, or displacement
	NOM: NR			
Qiu et al. (2016a) [18]	RF: AO standard plates + cancellous screws	FC	21 (100%)	NR
	NOM: NR			
Qiu (2016) [18]	RF: AO standard plates + cancellous screws	MRF	0 (0%)	NR
	NOM: NR			
Jayle et al. (2015) [51]	RF: titanium plates + screws	FC	10 (100%)	FC (≥ 3 fractures)
	NOM: NR			
Zhang Y (2015) [25]	RF: ORIF	FC with PC	24 (100%)	NR
	NOM: NR			
Zhang X (2015) [46]	RF: claw-type titanium plates	FC	23 (100%)	FC (≥ 3 fractures)
	NOM: standard conservative treatment			
Wada et al. (2015) [34]	RF: ORIF	FC + MRF	84 (100%)	NR
	NOM: NR			
Wu et al. (2015) [32]	RF: nickel–titanium alloy devices	FC + MRF	31 (41%)	FC (≥ 3 fractures), ≥ 3 rib fractures, dislocation, thorax deformity, or chest cavity active bleeding
	NOM: conservative management + chest strap			
Majercik et al. (2015) [16]	RF: plates + locking screws	FC + MRF	101 (75%)	FC, severely displaced fractures, intractable pain, failure to wean, or combination of these
	NOM: standard conservative management			
Xu et al. (2015) [28]	RF: titanium locking plates	FC	17 (100%)	NR
	NOM: standard conservative management			
Granhed and Pazooki (2014) [43]	RF: titanium plates + intramedullary splints	FC + MRF	56 (93%)	Impaired saturation in spite of oxygen administration; intractable pain
	NOM: NR			
Doben et al. (2014) [40]	RF: plates + intramedullary nails	FC	10 (100%)	Failure of nonoperative management
	NOM: standard conservative management			
Marasco et al. (2013) [50]	RF: inion resorbable plates + bicortical screws	FC	23 (100%)	FC (≥ 3 fractures) and ventilator dependent without prospect of weaning within 48 h
	NOM: mechanical ventilator management			
Khandelwal et al. (2011) [29]	RF: titanium plates + screws	FC + MRF	2 (5.3%)	NRS score > 7 on 10 days after trauma
	NOM: NR			
Moya et al. (2011) [31]	RF: titanium or steel plates	FC + MRF	9 (56%)	Intractable pain, ≥ 2 severely displaced rib fractures with pain, and respiratory failure
	NOM: NR			
Althausen et al. (2011) [26]	RF: locking plates + locking screws	FC	22 (100%)	FC with displacement, failure to wean, respiratory failure, or need of thoracotomy
	NOM: NR			
Solberg et al. (2009) [24]	RF: titanium plates	FC	9 (100%)	Superolateral chest wall deformity
	NOM: ventilatory pneumatic stabilization			
Nirula et al. (2006) [35]	RF: Adkin struts	FC + MRF	15 (50%)	FC, intractable pain, bleeding, and inability to wean
	NOM: NR			
Granetzny (2006) [23]	RF: K-wires and/or stainless steel wire	FC	20 (100%)	FC (≥ 3 rib fractures) with paradoxical chest wall movement
	NOM: strapping and packing			
Balci et al. (2004) [45]	RF: suture and traction	FC	27 (100%)	FC with paradoxical chest wall movement, respiratory failure, dyspnea, and insufficient blood gas
	NOM: endotracheal intubation			
Tanaka et al. (2002) [22]	RF: Judet struts	FC	18 (100%)	FC (≥ 6 fractures) with respiratory failure requiring mechanical ventilation and failure to wean
	NOM: internal pneumatic stabilization			
Voggenreiter (1996a) [19]	RF: ASIF reconstruction plates	FC without PC	10 (100%)	FC and thoracotomy for other injury, respiratory failure, paradoxical chest wall movement, or deformity
	NOM: standard conservative management			
Voggenreiter (1996a) [19]	RF: ASIF reconstruction plates	FC with PC	10 (100%)	FC and thoracotomy for other injury, respiratory failure, paradoxical chest wall movement, severe deformity
	NOM: standard conservative management			
Ahmed and Mohyuddin (1995) [37]	RF: K-wires	FC	26 (100%)	NR
	NOM: endotracheal intubation			
Kim et al. (1981) [49]	RF: Judet struts	FC	18 (100%)	NR
	NOM: internal pneumatic stabilization			
Aubert et al. (1981) [48]	RF: osteosynthesis	FC	22 (100%)	NR
	NOM: ventilator assistance, physiotherapy			

*RF* rib fixation, *NOM* nonoperative management, *NR* not reported, *FC* flail chest, *MRF* multiple rib fractures, *PC* pulmonary contusion

### Quality assessment

The average MINORS score of the included studies was 15.4 (SD 2.7; range 9–21). The MINORS score for RCTs was 20 (SD 1.0; range 19–21) and for observational studies 14.9 (SD 2.4; range 9–21). An overview of the study-specific MINORS score is provided in Appendix 3.

### Mortality

**Fig. 2:**
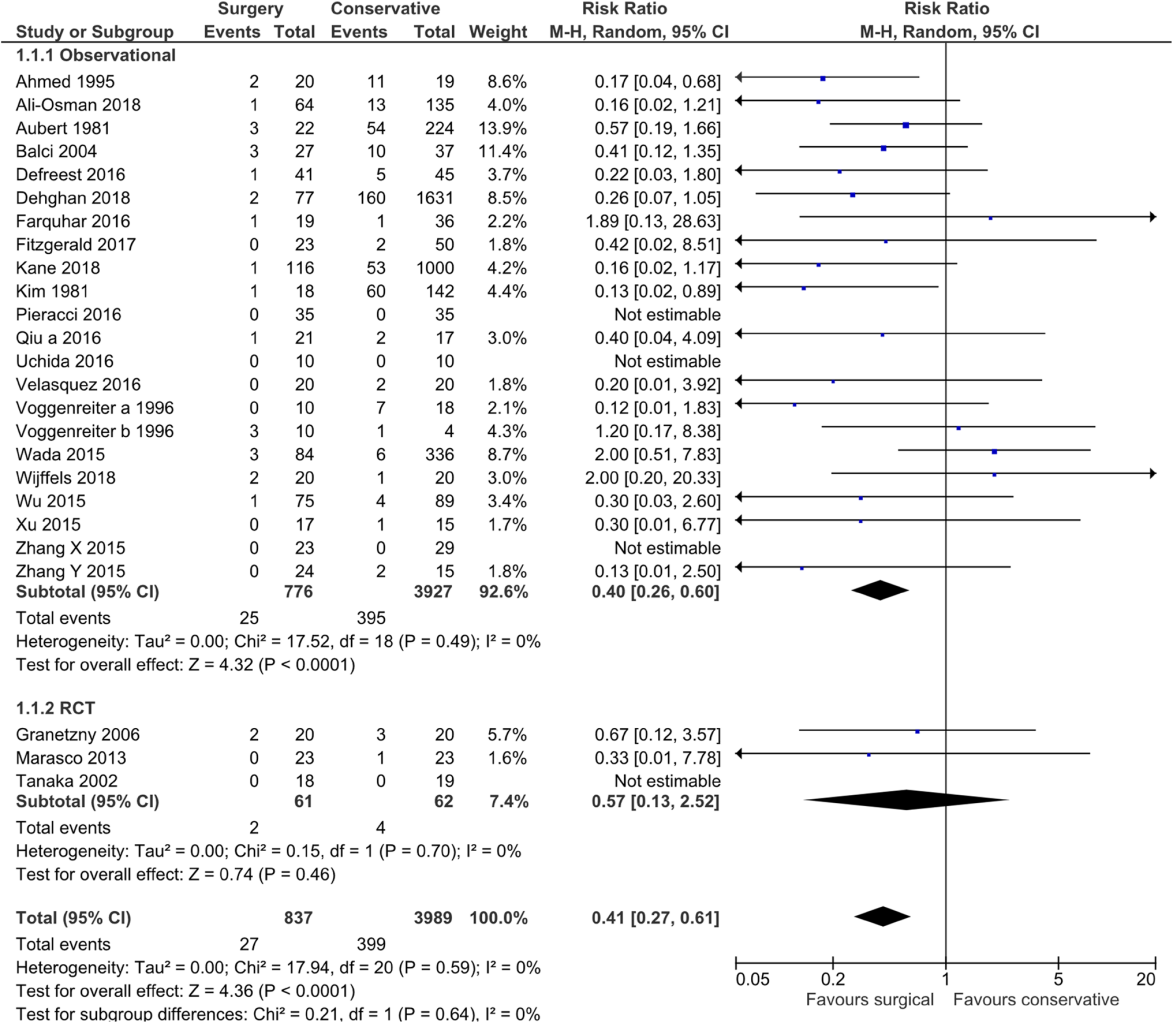
Mortality in a systematic review of rib fractures comparing operative to nonoperative treatment

**Fig. 3:**
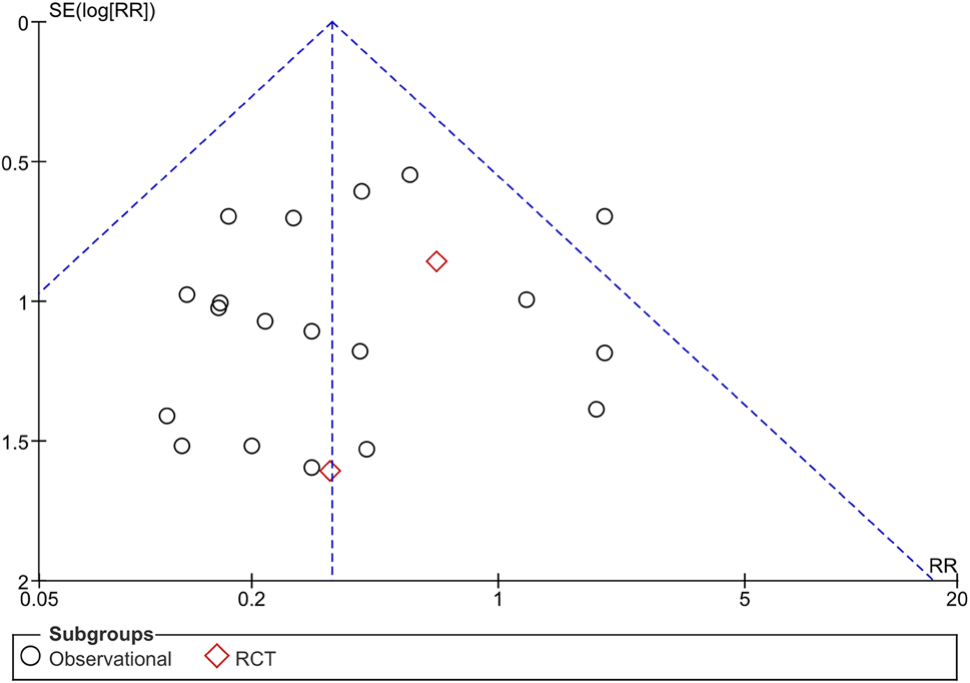
Funnel plot of studies included in a meta-analysis reporting mortality rates after operative or nonoperative treatment of rib fractures (*RR* risk ratio, *SE* standard error)

Twenty-five studies (*n* = 4826) reported on mortality (Online Appendix 4) [18, 19, 22, 23, 25, 27, 28, 30, 32–34, 36–50]. Rib fixation resulted in a significant reduction of mortality compared to nonoperative treatment with a risk ratio (RR) of 0.41 (95% CI 0.27, 0.61, *p* < 0.001, *I*^*2*^ = 0%) (Fig. 2). Different methods of incorporating studies in the meta-analysis with zero-event data in one or both arms yielded similar results (Online Appendix 5). When stratified by study design, RCTs showed a RR 0.57 (95% CI 0.13, 2.52, *p* = 0.46, *I*^*2*^ = 0%) vs. RR 0.40 (95% CI 0.26, 0.60, *p* < 0.001, *I*^*2*^ = 0%) in observational studies (Table 3). Figure 3 shows a funnel plot of the odds ratio and standard error of the included studies using the mortality rate; there was no important asymmetry observed.

**Table 3:** Subgroup and sensitivity analyses of studies included in a meta-analysis of rib fractures comparing rib fixation versus nonoperative treatment for patients with a flail chest

Analysis description	*n*	Mortality		*n*	HLOS		*n*	ILOS	
		RR (95% CI)	*P* value		MD (95% CI)	*P* value		MD (95% CI)	*P* value
All studies	25	0.41 (0.27, 0.61)	*p* < 0.001	21	− 1.46 (− 4.31, 1.39)	0.32	26	− 2.00 (− 3.61, − 0.38)	0.02
Subgroup analysis									
RCT	3	0.57 (0.13, 2.52)	0.46	2	− 8.33 (− 14.60, − 2.07)	0.009	3	− 6.37 (− 9.72, − 3.03)	*p* < 0.001
Observational studies	22	0.40 (0.26, 0.60)	*p* < 0.001	19	− 0.77 (− 3.72, 2.18)	0.61	23	− 1.53 (− 3.21, 0.15)	0.07
Sensitivity analysis									
High-quality studies	13	0.71 (0.35, 1.44)	0.34	15	− 3.53 (− 7.27, 0.21)	0.06	17	− 2.83 (− 4.75, − 0.91)	0.004
Studies after 2012	17	0.43 (0.25, 0.77)	0.004	16	− 0.64 (− 3.98, 2.69)	0.71	19	− 1.51 (− 3.40, 0.37)	0.12

Analysis description	*n*	DMV		*n*	Pneumonia		*n*	Tracheostomy	
		MD (95% CI)	*P* value		RR (95% CI)	*P* value		RR (95% CI)	*P* value
All studies	27	− 4.01 (− 5.58, − 2.45)	*p* < 0.001	25	0.59 (0.42, 0.83)	*p* <0.001	16	0.59 (0.39, 0.90)	0.01
Subgroup analysis									
RCT	3	− 5.88 (− 11.32, − 0.44)	0.03	3	0.36 (0.15, 0.85)	0.02	2	0.38 (0.14, 1.02)	0.05
Observational studies	23	− 3.79 (− 5.46, − 2.11)	*p* <0.001	22	0.63 (0.44, 0.92)	0.02	14	0.63 (0.40, 1.01)	0.05
Sensitivity analysis									
High-quality studies	17	− 3.87 (− 6.06, − 1.68)	0.000	16	0.55 (0.37, 0.82)	0.004	10	0.57 (0.41, 0.80)	0.001
Studies after 2012	18	− 3.27 (− 5.11, − 1.43)	0.000	16	0.73 (0.50, 1.06)	0.10	12	0.73 (0.47, 1.14)	0.16

*RCT* randomized controlled trial, *RR* risk ratio, *MD* mean difference, *CI* confidence interval, *n* no. of studies, *RR* risk ratio, *MD* mean difference

### Hospital stay length of stay

Twenty-one studies (*n* = 4770) reported on length of hospital stay (Online Appendix 4) [16, 17, 23, 25, 26, 31–35, 37–45, 47, 50, 51]. Rib fixation did not result in a significant reduction of HLOS compared to nonoperative treatment with a mean difference of −1.46 days (95% CI −4.31, 1.39, *p* = 0.32, *I*^2^ = 96%) (Online Appendix 6). When stratified by study design, the pooled mean difference of RCTs (−8.33 days; 95% CI −14.6, −2.1; *p* < 0.001, *I*^*2*^ = 46%) was greater compared to observational studies (−0.77; 95% CI −3.72, 2.18; *p* = 0.61, *I*^*2*^ = 97%) (Table 3).

### ICU length of stay

Twenty-six studies (*n* = 4520) reported on length of ICU stay (Online Appendix 4) [16–18, 22–26, 28, 30–33, 35–44, 47, 50, 51]. Rib fixation resulted in a significant reduction of ILOS compared to nonoperative treatment with a mean difference of −2.0 (95% CI −3.61, −0.38, *p* = 0.02, *I*^*2*^ = 85%) (Online Appendix 7). When stratified by study design, RCTs showed a greater difference compared to observational studies (Table 3).

### Duration of mechanical ventilation

Twenty-seven studies (*n* = 2063) reported on duration of mechanical ventilation (Online Appendix 4) [16–19, 22–28, 30–32, 35–42, 45–47, 49–51]. Rib fixation resulted in a significant reduction of days on mechanical ventilation compared to nonoperative treatment with a mean difference of −4.01 (95% CI −5.58, −2.45, *p* < 0.001, *I*^*2*^ = 91%) (Online Appendix 8). When stratified by study design, RCTs showed a greater difference compared to observational studies (Table 3).

### Pneumonia

Twenty-five studies (*n* = 4485) reported on the incidence of pneumonia (Online Appendix 4) [16–19, 22, 24–26, 28, 30–33, 37–39, 41–44, 47, 50, 51]. Rib fixation resulted in a significant reduction of pneumonia compared to nonoperative treatment with a risk ratio of 0.59 (95% CI 0.42, 0.83, *p* = 0.002, *I*^*2*^ = 79%) (Online Appendix 9). When stratified by study design both subgroups showed similar results (Table 3).

### Tracheostomy

Fourteen studies (*n* = 1541) reported on the need of tracheostomy (Online Appendix 4) [16–18, 22, 25, 26, 28, 30, 32, 34, 36–38, 45, 50]. Rib fixation resulted in a significant reduction of tracheostomies compared to nonoperative treatment with a risk ratio of 0.59 (95% CI 0.36, 0.90, *p* = 0.01, *I*^*2*^ = 72%) (Online Appendix 10). When stratified by study design both subgroups showed similar results (Table 3).

### Other outcome measures

Nine studies (*n* = 1174) reported on implant removal; five studies reported zero events and four studies reported implant removal ranging from 1.5 to 4.9% (Online Appendix 4) [17, 26, 28, 36–38, 40, 45, 48]. Eleven studies reported on wound infection; five studies reported zero events and six studies reported a wound infection rate ranging from 1.7 to 25% [18, 23, 24, 26–30, 46]. Other short and/or long-term complications were poorly reported and described mainly respiratory complications.

### Sensitivity analyses

In sensitivity analysis for study quality, results did not change significantly except for HLOS which increased in favor of rib fixation in studies with higher quality with a mean difference of –3.53 (95% CI −7.27, −0.21, *p* = 0.06) (Table 3). Results from studies published after 2012 did not show a reduced HLOS, ILOS, incidence of pneumonia or need for tracheostomy after rib fixation (Table 3).

## Discussion

In this systematic review and meta-analysis of RCTs and observational studies, rib fixation for patients with flail chest resulted in lower mortality, shorter ILOS and DMV, lower pneumonia rate, and lower need for tracheostomy. Pooled results from RCTs and observational studies were similar for all studied outcome measures although results from RCTs showed a larger treatment effect for HLOS, ILOS, and DMV. Results from recent studies showed lower mortality and shorter DMV after rib fixation, but there were no significant differences for the other outcome measures. The implant removal rate ranged from 1.5 to 4.9%. There were not enough studies of only patients with multiple rib fractures to perform meta-analyses on rib fixation for this patient population.

This meta-analysis included a large number of studies demonstrating the potential short-term benefit of rib fixation over nonoperative treatment for flail chest. Most often the indication for rib fixation was the presence of flail chest and to a lesser extent respiratory failure or intractable pain. Even though almost all studies included patients with flail chest, in many cases it was unclear whether it was a radiological or clinical flail chest making results harder to interpret. It is important to distinguish between these subgroups as respiratory compromise as well as injury severity is thought to mark important differences and influence outcome. The heterogeneous indication and patient populations reported on in the literature mask the exact indication and patient subgroup that would benefit most from rib fixation and consequently the adaptation of rib fixation in current practice.

Very few studies are available investigating patients with multiple rib fractures without flail chest. In a retrospective study, Qiu et al. performed separate analysis on patients with multiple rib fractures without flail segment and showed good short-term results and an earlier return to ‘normal activity’ after rib fixation [18]. Another notable study on multiple rib fractures was from Khandelwal et al. who described a prospective cohort of patients with multiple rib fractures where most patients had two or three rib fractures and only two (5.3%) had a flail chest [29]. They reported a significant reduction of pain and earlier return to work after rib fixation. No other studies have reported on rib fixation compared to nonoperative treatment focused on multiple rib fractures even though this is the largest subgroup of patients seen in daily practice.

In this review, we have included both RCTs and observational studies and show similar results for all outcome measures between both designs. Concato et al., Benson et al., and Ioannides et al. have provided an empirical basis for the comparison of RCTs and observational studies and showed results from these different designs can be remarkably similar, but can be rather different as well [52–54]. Although, treatment effects can be similar across studies regardless of design, genuine differences in treatment effects between different patient populations may be masked by biases in observational studies. Pooling results across different design could then lead to incorrect inferences. The judgement about validity of pooling results from different designs should be made on a case-by-case basis, since for instance the potential for confounding bias is context- and research-specific. Still, within the field of (orthopedic) trauma surgery there is growing evidence showing the potential of observational studies in meta-analyses leading to more robust conclusions without decreasing quality of the results [7–9].

Interestingly, RCTs in this study showed a larger treatment effect for some of the outcome measures as compared to observational studies. It is thought that observational studies tend to overestimate treatment effect which is possibly the result of the surgeon introducing a selection bias by choosing the optimal patient or publication bias [55, 56]. The three RCTs available on this subject all had very strict inclusion and exclusion criteria resulting in specific patient groups where treatment effects could be demonstrated yet with limited generalizability [22, 23, 50]. In observational studies, usually with less strict inclusion and exclusion criteria, an unclear indication together with other serious concomitant injuries can result in a selection of patients including patients who would benefit more from nonoperative treatment. A wrong patient selection can reduce measured treatment effects after rib fixation which could explain differences found between RCTs and observational studies in this specific topic. Additionally, differences in timing of the surgical procedure between studies might have introduced bias in comparability as early surgical stabilization is associated with favorable outcomes [57]. However, data regarding timing of surgery were not sufficiently reported in the included studies to further explore these effects. Finally, improvement of intensive care management over time could have attributed to differences in treatment effects as shown by our sensitivity analysis. In more recent studies only mortality and DMV improved after rib fixation, but there was no difference for the other outcome measures.

This study had some limitations. First, the results may be altered by missed studies in the literature search or by publication bias. However, we performed an extensive search using multiple databases with citation and reference checking of included studies. A funnel plot of the primary outcome measure did not suggest bias due to selective publication. Therefore, we are confident that we have a representative overview of the current literature. Second, we did not distinguish between studies with both flail chest and multiple rib fractures and studies including only flail chest patients. Very few patients with multiple rib fractures were included in these studies. Therefore, we think results from these studies translate to flail chest patients and should not be excluded from analyses. Still, cautious interpretation of study results is necessary as the variety of definitions used in the included studies might have resulted in a high in-between study variability of patient samples.

More research is needed to further identify the right indication and right patient for rib fixation. As previously mentioned, RCTs in this heterogenic population are very difficult to perform and for adequate subgroup analyses sufficiently large sample sizes are needed. In the rapidly developing area of surgery, RCTs can be expensive, time consuming, and often have limitations in terms of generalizability and small sample sizes due to strict inclusion and exclusion criteria [58, 59]. Observational studies show similar results as compared to RCTs and might be an achievable first step in gathering high-quality evidence. Currently a large prospective multicenter database is created in the Netherlands including both patients with flail chest and multiple rib fractures from multiple level-1 trauma centers, aiming to answer the above questions with the use of large sample sizes and long-term follow-up [60].

## Conclusion

Rib fixation significantly improves short-term outcome for patients with flail chest, although the indication and patient subgroup who would benefit most from this treatment remain unclear. There is not enough data regarding patients with multiple rib fractures without flail segment. Observational studies show similar results as compared to RCTs and might be an achievable first step in gathering high-quality evidence. Larger prospective studies are required to investigate proper indications and relevant outcome after rib fixation.

## Electronic supplementary material

Below is the link to the electronic supplementary material.

Supplementary material 1 (DOCX 9071 KB)
