# Supplementary material for: Fixation of flail chest or multiple rib fractures: current evidence and how to proceed. A systematic review and meta-analysis
Source: Eur J Trauma Emerg Surg. 2018 Oct 1;45(4):631–44. doi: 10.1007/s00068-018-1020-x (PMC6689030; doi:10.1007/s00068-018-1020-x)

**Appendix 1.** Search Syntax

Date searched: June 16^th^ 2018

Search string Pubmed (n=698 )

(“Rib Fractures”[Mesh] OR rib fracture* OR “flail chest”[Mesh]) AND (surgical management OR fixation OR plating OR orif)

Search string Embase (n=847)

(‘rib fracture’/exp OR (rib NEAR/1 fracture*):ab,ti OR ‘flail chest’:ab,ti) AND (‘fracture treatment’/exp OR orif:ab,ti OR fixation:ab,ti OR plating:ab,ti)

Search string CENTRAL (n=195)

(“rib fracture*” OR “flail chest”)

Search string CINAHL (n=612)

(''rib fracture*")

| **Criteria** | **2** | **1** | **0** |
| --- | --- | --- | --- |
| **A clearly stated aim** | Aim or hypothesis including outcomes have been reported | Aim or hypothesis have been reported without a clear outcome | Not reported |
| **Inclusion of consecutive patients** | Explicit inclusion and exclusion criteria have been reported | Unclear or poor description inclusion and exclusion criteria have been reported | Not reported |
| **Prospective collection of data** | Prospective | Retrospective | Not reported |
| **Endpoints appropriate to the aim of the study** | Outcomes are appropriate to the aim of the study | Outcomes are not appropriate to the aim of the study | Not reported |
| **Unbiased assessment of the study endpoint** | Blind evaluation of objective outcomes and double-blind evaluation of subjective outcomes | One or more outcomes have been blinded | No blinding / not reported |
| **Follow-up period appropriate to the aim of the study** | ≥ 1 year | < 1 year | Not reported |
| **Loss to follow-up less than 5%** | ≤ 5% | > 5% and ≤ 20% | Not reported / >20% |
| **Prospective calculation of the study size** | Power analysis has been performed | Explanation for the number of included patients without a power analysis | Not reported / not performed |
| **An adequate control group** | Plate or intramedullary fixation compared with a conservative treatment | Not applicable | Not reported |
| **Contemporary groups** | Study group and controls have been managed during the same time period | Study group and controls have not been managed during the same time period | Not reported / unclear discription |
| **Baseline equivalence of groups** | Baseline characteristics have been described for both groups and are comparable | Baseline characteristics have not been described thoroughly or are not comparable | Not reported |
| **Adequate statistical analyses** | Statistical analysis has been described including the type of test | Inadequate statistical analysis | Not reported |

**Appendix 2.** MINORS assessment criteria

**Appendix 3.** Quality assessment of all included studies in a systematic review of proximal humerus fractures comparing operative to nonoperative treatment.

| **Criteria** | Aubert 1981 | Kim 1981 | Ahmed 1995 | Voggenreiter 1996 | Tanaka 2002 | Balci 2004 | Granetzny 2005 | Nirula 2006 | Solberg 2009 | Althausen 2011 | Moya 2011 | Khandelwal 2011 | Marasco 2013 | Doben 2014 | Granhed 2014 | Xu 2015 | Majercik a + b 2015 | Wu 2015 | Wada 2015 | Zhang X 2015 | Zhang Y 2015 | Jayle 2015 | Qiu 2016 | Velasquez 2016 | Uchida 2016 | DeFreest 2016 | Pieracci 2016 | Farquhar 2016 | Fitzgerald 2017 | Dehghan 2018 | Ali-Osman 2018 | Wijffels 2018 | Kane 2018 |
| --- | --- | --- | --- | --- | --- | --- | --- | --- | --- | --- | --- | --- | --- | --- | --- | --- | --- | --- | --- | --- | --- | --- | --- | --- | --- | --- | --- | --- | --- | --- | --- | --- | --- |
| A Clearly stated aim | 2 | 2 | 2 | 2 | 2 | 2 | 2 | 2 | 1 | 2 | 2 | 1 | 2 | 2 | 2 | 2 | 2 | 1 | 2 | 2 | 2 | 2 | 2 | 2 | 2 | 2 | 2 | 2 | 2 | 2 | 2 | 2 | 2 |
| Inclusion of consecutive patients | 2 | 1 | 2 | 2 | 2 | 2 | 2 | 0 | 2 | 2 | 1 | 2 | 2 | 2 | 2 | 2 | 2 | 2 | 2 | 1 | 2 | 2 | 2 | 2 | 2 | 2 | 2 | 2 | 2 | 2 | 2 | 2 | 0 |
| Prospective collection of data | 1 | 1 | 1 | 1 | 2 | 1 | 2 | 1 | 1 | 1 | 1 | 2 | 2 | 1 | 1 | 1 | 1 | 2 | 1 | 1 | 1 | 1 | 1 | 1 | 1 | 1 | 2 | 1 | 0 | 0 | 0 | 0 | 0 |
| Endpoints appropriate to the aim of the study | 2 | 2 | 2 | 2 | 2 | 2 | 2 | 2 | 2 | 2 | 2 | 1 | 2 | 2 | 2 | 2 | 2 | 2 | 2 | 2 | 2 | 2 | 2 | 2 | 2 | 2 | 2 | 2 | 2 | 1 | 2 | 2 | 1 |
| Unbiased assessment of the study endpoint | 0 | 0 | 0 | 0 | 0 | 0 | 0 | 0 | 0 | 0 | 0 | 2 | 0 | 0 | 0 | 0 | 0 | 0 | 0 | 0 | 0 | 0 | 0 | 0 | 0 | 0 | 0 | 0 | 0 | 0 | 0 | 0 | 0 |
| Follow-up period appropriate to the aim of the study | 2 | 1 | 1 | 1 | 2 | 0 | 2 | 1 | 2 | 2 | 2 | 2 | 2 | 2 | 2 | 2 | 2 | 2 | 2 | 2 | 2 | 2 | 2 | 2 | 2 | 2 | 2 | 2 | 1 | 2 | 2 | 2 | 2 |
| Loss to follow-up less than 5% | 1 | 1 | 2 | 0 | 2 | 0 | 2 | 0 | 0 | 0 | 2 | 2 | 1 | 2 | 0 | 0 | 2 | 0 | 0 | 0 | 1 | 0 | 0 | 2 | 0 | 2 | 1 | 2 | 1 | 0 | 1 | 1 | 0 |
| Prospective calculation of the study size | 0 | 0 | 0 | 0 | 0 | 0 | 0 | 0 | 0 | 2 | 0 | 0 | 2 | 0 | 0 | 0 | 0 | 0 | 0 | 0 | 0 | 0 | 0 | 0 | 0 | 0 | 0 | 0 | 0 | 0 | 0 | 0 | 0 |
| An adequate control group | 0 | 0 | 0 | 0 | 2 | 1 | 2 | 2 | 1 | 1 | 1 | 1 | 2 | 1 | 1 | 0 | 2 | 2 | 2 | 2 | 0 | 2 | 0 | 1 | 2 | 1 | 2 | 1 | 2 | 1 | 1 | 2 | 1 |
| Contemporary groups | 1 | 1 | 1 | 2 | 2 | 1 | 2 | 2 | 2 | 2 | 1 | 2 | 2 | 1 | 0 | 2 | 2 | 2 | 2 | 2 | 2 | 1 | 1 | 1 | 2 | 1 | 1 | 1 | 1 | 1 | 1 | 1 | 1 |
| Baseline equivalence of groups | 0 | 0 | 1 | 2 | 2 | 1 | 1 | 1 | 2 | 2 | 2 | 0 | 2 | 2 | 0 | 2 | 1 | 2 | 2 | 2 | 2 | 2 | 2 | 2 | 2 | 2 | 1 | 1 | 2 | 1 | 1 | 2 | 1 |
| Adequate statistical analyses | 2 | 2 | 0 | 2 | 2 | 2 | 2 | 2 | 2 | 2 | 2 | 2 | 2 | 2 | 2 | 2 | 2 | 2 | 2 | 2 | 2 | 2 | 2 | 2 | 2 | 2 | 2 | 2 | 2 | 1 | 2 | 2 | 1 |
| **Total quality score MINORS** | **13** | **11** | **12** | **14** | **20** | **12** | **19** | **13** | **15** | **18** | **16** | **17** | **21** | **17** | **12** | **15** | **18** | **17** | **17** | **16** | **16** | **16** | **14** | **17** | **17** | **17** | **17** | **16** | **15** | **11** | **14** | **16** | **9** |
|  |  |  |  |  |  |  |  |  |  |  |  |  |  |  |  |  |  |  |  |  |  |  |  |  |  |  |  |  |  |  |  |  |  |

| **Table 2. Results of the included studies comparing operative versus non-operative management of traumatic rib fractures.** | | | | | | | |
| --- | --- | --- | --- | --- | --- | --- | --- |
|  |  |  |  |  |  |  |  |
| Study | Treatment groups | Mortality | Hospital LOS (days) | ICU LOS (days) | Duration of mechanical ventilation (days) | Pneumonia | Tracheostomy |
|  |  |  |  |  |  |  |  |
|  |  |  |  |  |  |  |  |
| Dehghan 2018 | Operative | 2 (2.6%) | 21 ± 20 | 15 ± 13 | NR | 45 (48%) | 17 (22%) |
|  | Non-operative | 160 (9.8%) | 17 ± 26 | 13 ± 15 |  | 614 (38%) | 182 (11%) |
| Ali-Osman 2018 | Operative | 1 (1.6%) | 12 [9-16] | 6 [3-10] | 3 [1-15] | 5 (7.8%) | NR |
|  | Non-operative | 13 (9.6%) | 4.8 [2.9-8.4] | 4 [3-7] | 4 [1-10] | 16 (12%) |  |
| Wijffels 2018 | Operative | 2 (10%) | 21 [12-33] | 5 [3-13] | 4 [2-10] | 7 (35%) | NR |
|  | Non-operative | 1 (5%) | 23 [17-42] | 12 [3-29] | 18 [12-26] | 16 (80%) | NR |
| Kane 2018 | Operative | 1 (0.9%) | 12 [10-14] | 3 [0-6] | NR | 7 (6%) | 10 (8.6%) |
|  | Non-operative | 13 (1.3%) | 5 [3-9] | 0 [0-3] |  | 59 (6%) | 45 (4.5%) |
| Fitzgerald 2017 | Operative | 0 (0%) | 18 (14-23) | 12 (7-17) | NR | 0 (0%) | NR |
|  | Non-operative | 2 (4%) | 17 (10-23) | 8 (5-11) |  | 7 (14%) |  |
| Farquhar 2016 | Operative | 1 (5,3%) | 21.9 + 13.2 | 7.4 + 6.7 | 6.1 + 5.9 | 12 (63%) | NR |
|  | Non-operative | 1 (2,8%) | 16.0 + 12.1 | 3.7 + 6.0 | 3.1 + 5.5 | 8 (22%) |  |
| Pieracci 2016 | Operative | 0 (0%) | 13.0 [9.0, 21.0] | 6.0 [3.0, 10.0] | 0 [0.0, 8.0] | 7 (20%) | 5 (14%) |
|  | Non-operative | 0 (0%) | 16.0 [10.0, 23.0] | 9.0 [4.0, 15.0] | 5.0 [0, 18] | 11 (31%) | 16 (46%) |
| Defreest 2016 | Operative | 1 (2,4%) | 28.3 (9-69) | 14.0 (0-43) | 9.3 (0-39) | 11 (27%) | 10 (24%) |
|  | Non-operative | 5 (11,1%) | 13.0 (3-43) | 8.0 (0-43) | 5.8 (0-39) | 10 (22%) | 8 (18%) |
| Uchida 2016 | Operative | 0 (0%) | NR | 6.5 [3, 9] | 5.5 [1, 8] | 2 (20%) | 1 (10%) |
|  | Non-operative | 0 (0%) |  | 12 [8, 14] | 9 [7, 12] | 9 (90%) | 3 (30%) |
| Velasquez 2016 | Operative | 0 (0%) | 6 [4, 10] | 4.5 [1, 8] | 2 [1, 3] | 3 (15%) | NR |
|  | Non-operative | 2 (10%) | 16 [11, 22] | 8 [6, 10.5] | 10 [6, 16] | 13 (65%) |  |
| Qiu a 2016 | Operative | 1 (4,8%) | NR | 7.2 + 1.7 | 5.7 + 1.4 | NR | 2 (9,5%) |
|  | Non-operative | 2 (11,8%) |  | 10.3 + 2.3 | 9.1 + 3.6 |  | 8 (47%) |
| Qiu b 2016 | Operative | 0 (0%) | 11.1 + 1.9 | NR | NR | 3 (4,6%) | NR |
|  | Non-operative | 0 (0%) | 15.9 + 2.8 |  |  | 10 (17%) |  |
| Jayle 2015 | Operative | NR | 21.7 + 7.8 | 9.0 + 4.3 | 3.1 + 5.2 | 4 (40%) | NR |
|  | Non-operative | NR | 32.3 + 19.3 | 12.3 + 8.5 | 5.9 + 9.4 | 3 (30%) |  |
| Zhang Y 2015 | Operative | 0 (0%) | 38 [33, 54.25] | 4.5 [21.3, 30.7] | 12 [7.5, 17.8] | 16 (67%) | 12 (50%) |
|  | Non-operative | 2 (13,3%) | 60 [38, 99.75] | 21.5 [18, 33.5] | 7 [4, 14] | 7 (47%) | 7 (9,7%) |
| Zhang X 2015 | Operative | 0 (0%) | NR | 5.5 + 6.4 | 4.1 + 6.1 | NR | NR |
|  | Non-operative | 0 (0%) |  | 14.2 + 6.5 | 14 + 7.6 |  |  |
| Wada 2015 | Operative | 3 (3,6%) | 33 [22, 45] | NR | NR | NR | 10 (12%) |
|  | Non-operative | 6 (1,8%) | 42 [23, 58] |  |  |  | 68 (20%) |
| Wu 2015 | Operative | 1 (1,3%) | 15.3 + 6.4 | 8.2 + 4.3 | 3.7 + 1.4 | 5 (6,7%) | 4 (5,3%) |
|  | Non-operative | 4 (4,5%) | 26.5 + 6.9 | 14.6 + 3.2 | 9.5 + 4.3 | 17 (19%) | 7 (7,9%) |
| Majercik 2015 | Operative | NR | 11.4 + 5.7 | 4.6 + 5.6 | 0 [0, 3] | 12 (8,8%) | 8 (5,8%) |
|  | Non-operative |  | 12.3 + 9.1 | 5.9 + 7.7 | 0 [0, 4] | 55 (20%) | 30 (11%) |

**(table continued)**

| Xu 2015 | Operative | 0 (0%) | NR | 15.9 + 5.0 | 10.5 + 3.7 | 10 (59%) | 2 (12%) |
| --- | --- | --- | --- | --- | --- | --- | --- |
|  | Non-operative | 1 (6,7%) |  | 19.6 + 5.0 | 13.7 + 4.4 | 12 (93%) | 6 (40%) |
| Granhed 2014 | Operative | 2 (3,3%) | NR | NR | 2.7 (0-21) | 0 (0%) | NR |
|  | Non-operative | NR |  |  | 9.0 (1-76) | NR |  |
| Doben 2014 | Operative | N/A | 21.6 (8-59) | 12.5 (5-21) | 8.2 (0-30) | NR | NR |
|  | Non-operative | 0 (0%) | 28.5 (6-50) | 15.3 (5-22) | 18.0 (4-40) |  |  |
| Marasco 2013 | Operative | 0 (0%) | 20 [18, 28] | 13.5 [9.9, 15.8] | 6.3 + 3.4 | 11 (48%) | 9 (3,9%) |
|  | Non-operative | 1 (4,3%) | 25 [18, 38] | 18.7 [13.4, 26.9] | 7.5 + 5.4 | 17 (74%) | 16 (7,0%) |
| Khandelwal 2011 | Operative | NR | NR | NR | NR | NR | NR |
|  | Non-operative |  |  |  |  |  |  |
| Moya 2011 | Operative | NR | 18 + 12 | 9 + 8 | 7 + 8 | 5 (31%) | NR |
|  | Non-operative |  | 16 + 11 | 7 + 10 | 6 + 10 | 12 (38%) |  |
| Althausen 2011 | Operative | NR | 11.9 + 7.8 | 7.6 + 7.4 | 4.2 + 6.6 | 1 (4,5%) | 3 (3,9%) |
|  | Non-operative |  | 19.0 + 12.6 | 9.7 + 9.2 | 9.7 + 9.2 | 7 (25%) | 11 (3,9%) |
| Solberg 2009 | Operative | NR | NR | 5.4 + 1.5 | 1.9 + 1.1 | 0 (0%) | NR |
|  | Non-operative |  |  | 21 + 13.6 | 13.3 + 5.3 | 3 (43%) |  |
| Nirula 2006 | Operative | NR | 18.8 + 1.8 | 12.1 + 1.2 | 6.5 + 1.3 | NR | NR |
|  | Non-operative |  | 21.1 + 3.9 | 14.1 + 2.7 | 11.2 + 2.6 |  |  |
| Granetzny 2006 | Operative | 2 (10%) | 11.7 + 10.1 | 9.6 + 12.0 | 2 + 8.9 | NR | NR |
|  | Non-operative | 3 (15%) | 23.1 + 10.1 | 14.6 + 12.0 | 12 + 8.9 |  |  |
| Balci 2004 | Operative | 3 (1,11%) | 18.3 + 7.6 | NR | 3.1 + 1.8 | NR | 0 (0%) |
|  | Non-operative | 10 (27,0%) | 19.2 + 7.2 | NR | 7.2 + 5.8 |  | 7 (19%) |
| Tanaka 2002 | Operative | 0 (0%) | NR | 16.5 + 7.4 | 10.8 + 3.4 | 4 (22%) | 3 (17%) |
|  | Non-operative | 0 (0%) |  | 26.8 + 13.2 | 18.3 + 7.4 | 17 (90%) | 15 (79%) |
| Voggenreiter a 1996 | Operative | 0 (0%) | NR | NR | 6.5 + 7.0 | 1 (10%) | NR |
|  | Non-operative | 7 (38,9%) |  |  | 26.7 + 29.0 | 5 (28%) |  |
| Voggenreiter b 1996 | Operative | 3 (30%) | NR | NR | 30.8 + 33.7 | 4 (40%) | NR |
|  | Non-operative | 1 (25%) |  |  | 29.3 + 22.5 | 2 (50%) |  |
| Ahmed 1995 | Operative | 2 (10%) | NR | 9 | 3.9 | NR | 3 (15%) |
|  | Non-operative | 11 (57,9%) |  | 21 | 15 |  | 14 (74%) |
| Kim 1981 | Operative | 1 (5,9%) | NR | NR | 24 + 15 | NR | NR |
|  | Non-operative | 60 (42,2%) |  |  | 22.1 + 13.5 | 7 (4,9%) |  |
| Aubert 1981 | Operative | 3 (13,6%) | NR | NR | NR | NR | NR |
|  | Non-operative | 54 (24,1%) |  |  |  |  | 135 (60%) |

| **Appendix 5.** Impact of different methods to handle zero-event data in a meta-analysis of operative versus nonoperative treatment of rib fractures and mortality | | | |
| --- | --- | --- | --- |
| Method |  |  |  |
|  | Observational studies | RCT | Total |
|  | OR (95% CI) | OR (95% CI) | OR (95% CI) |
| Mantel-Haenzel* | 0.43 (0.27 – 0.69) | 0.57 (0.13 – 2.52) | 0.44 (0.28 – 0.69) |
| Crude | 0.21 (0.13 – 0.35) | 0.49 (0.09 – 2.79) | 0.22 (0.14 – 0.35) |
| Inverse variance - no correction | 0.41 (0.23 – 0.73) | 0.63 (0.09 – 4.24) | 0.43 (0.25 – 0.74) |
| Inverse variance - with correction | 0.39 (0.23 – 0.65) | 0.59 (0.13 – 2.68) | 0.41 (0.25 – 0.66) |
| DerSimonian Laird with correction | 0.37 (0.17 – 0.79) | 0.58 (0.11 – 3.23) | 0.39 (0.20 – 0.78) |
| Peto | 0.28 (0.16 – 0.49) | 0.50 (0.07 – 3.47) | 0.29 (0.17 – 0.50) |
| * Method used in meta-analysis; OR odds-ratio; CI confidence interval | | |  |
| In a model with correction 0.5 is added to every table of the 2x2 table | | |  |

**Appendix 6.** Hospital length of stay in a systematic review of rib fractures comparing operative to nonoperative treatment


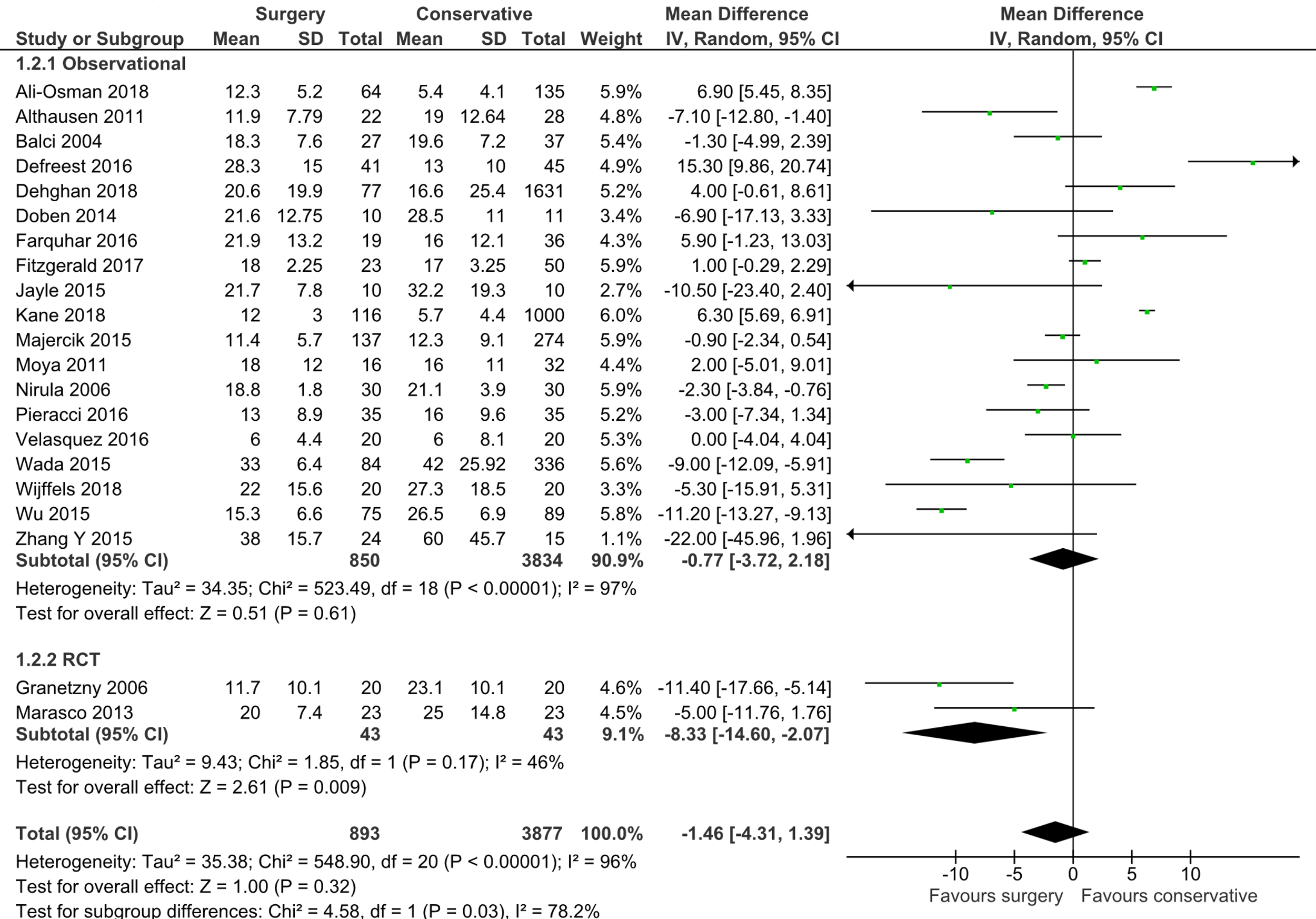


**Appendix 7.** Intensive care length of stay in a systematic review of rib fractures comparing operative to nonoperative treatment


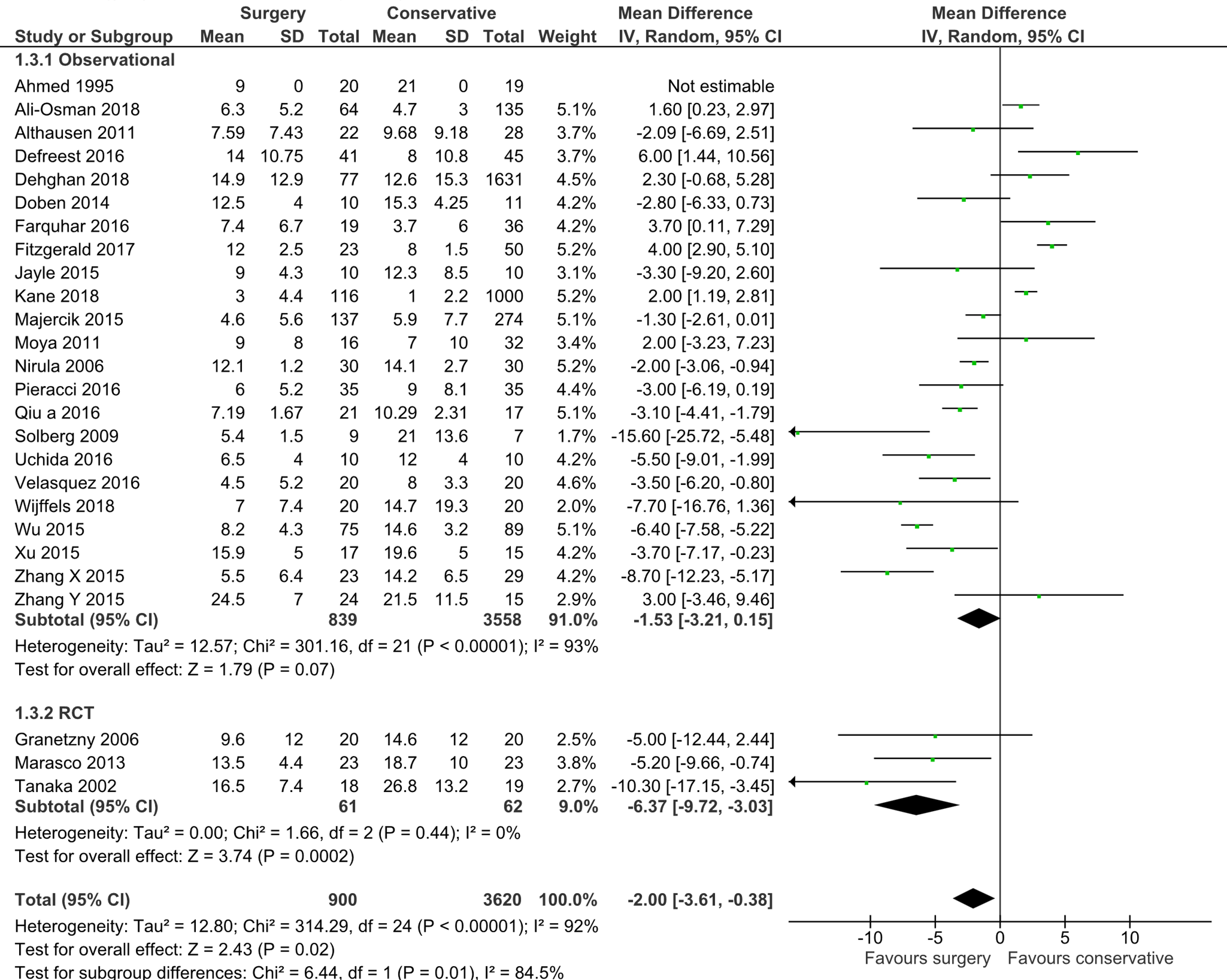


**Appendix 8.** Duration of mechanical ventilation in a systematic review of rib fractures comparing operative to nonoperative treatment


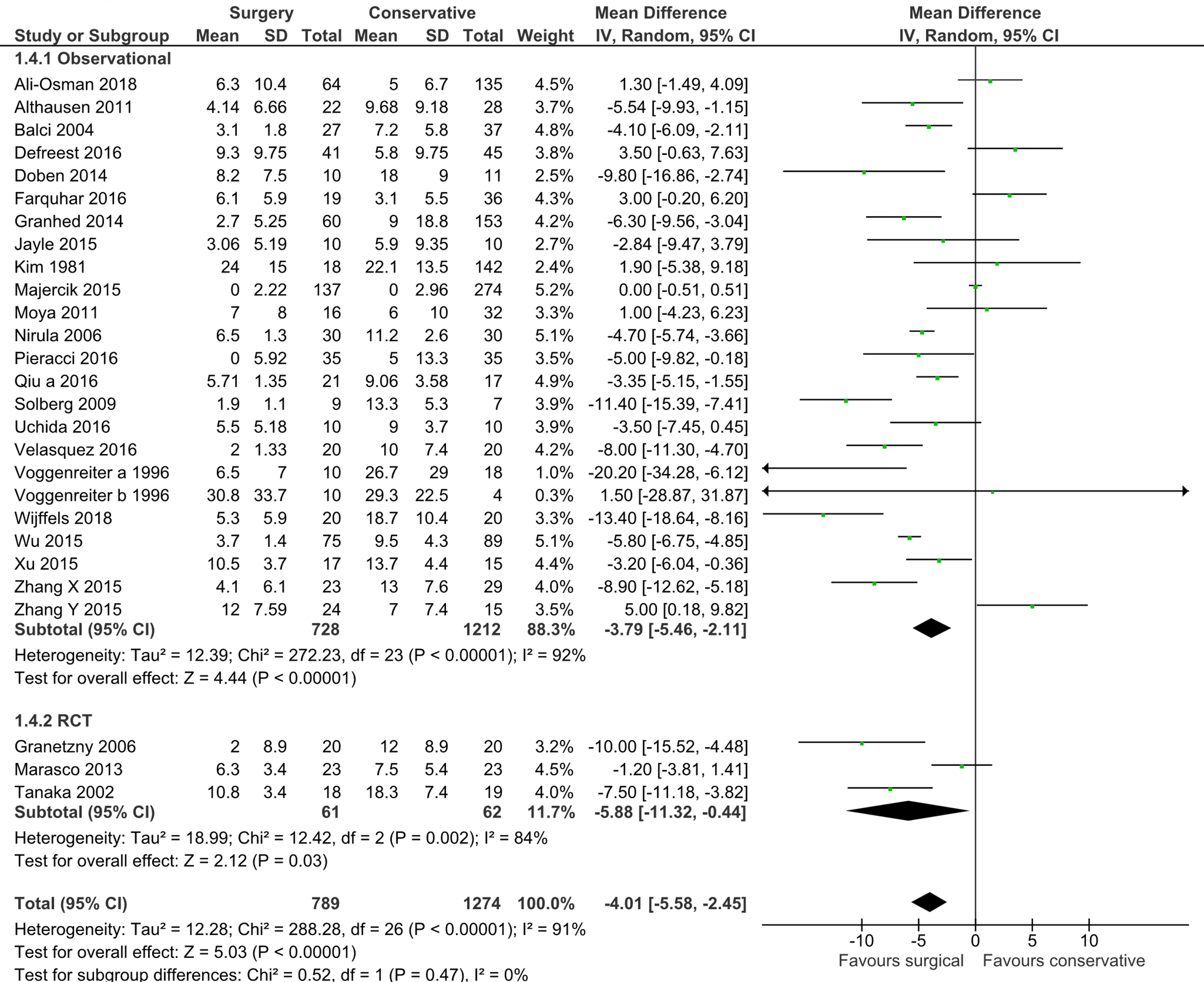


**Appendix 9.** Pneumonia in a systematic review of rib fractures comparing operative to nonoperative treatment


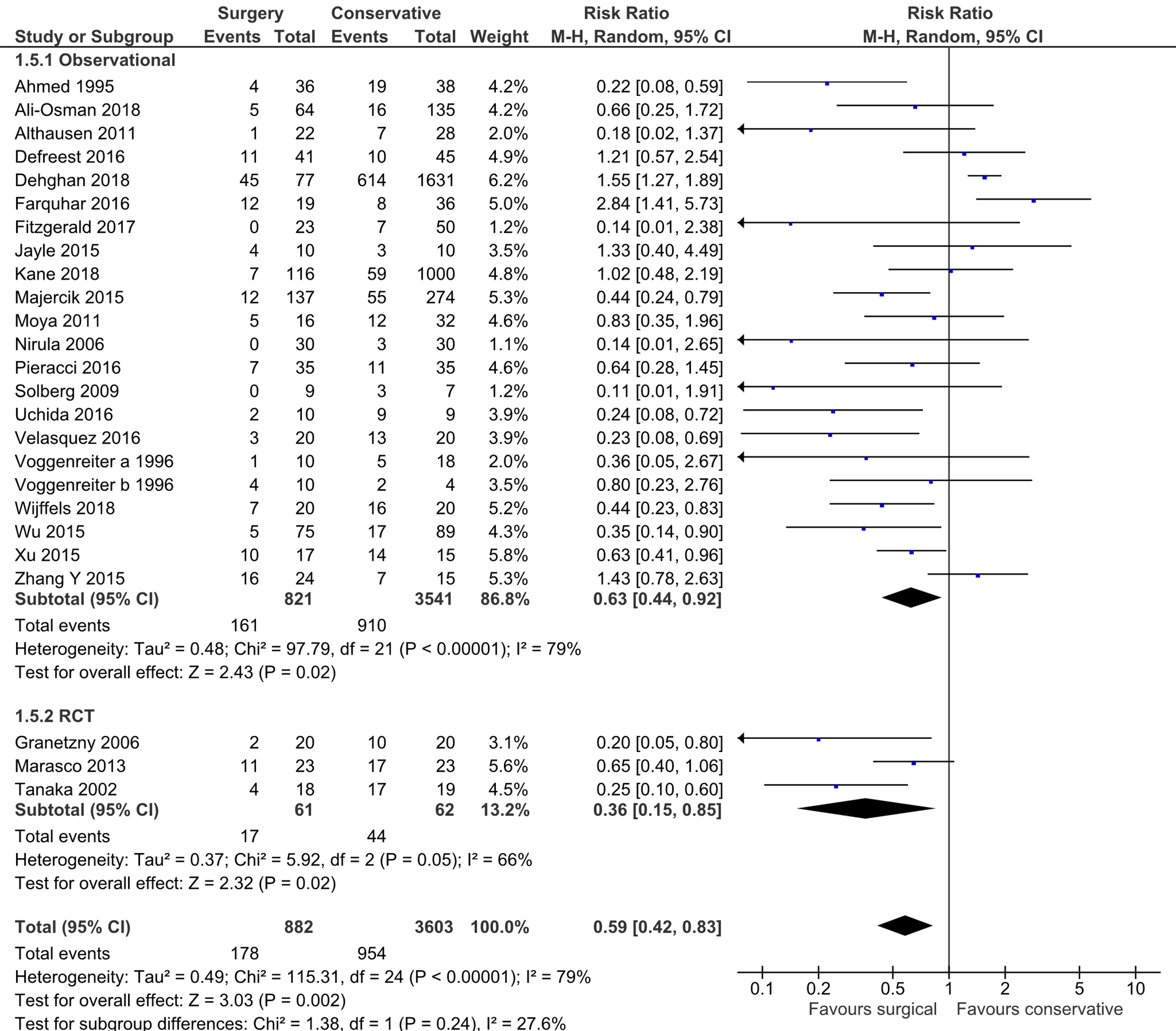


**Appendix 10.** Tracheostomy in a systematic review of rib fractures comparing operative to nonoperative treatment


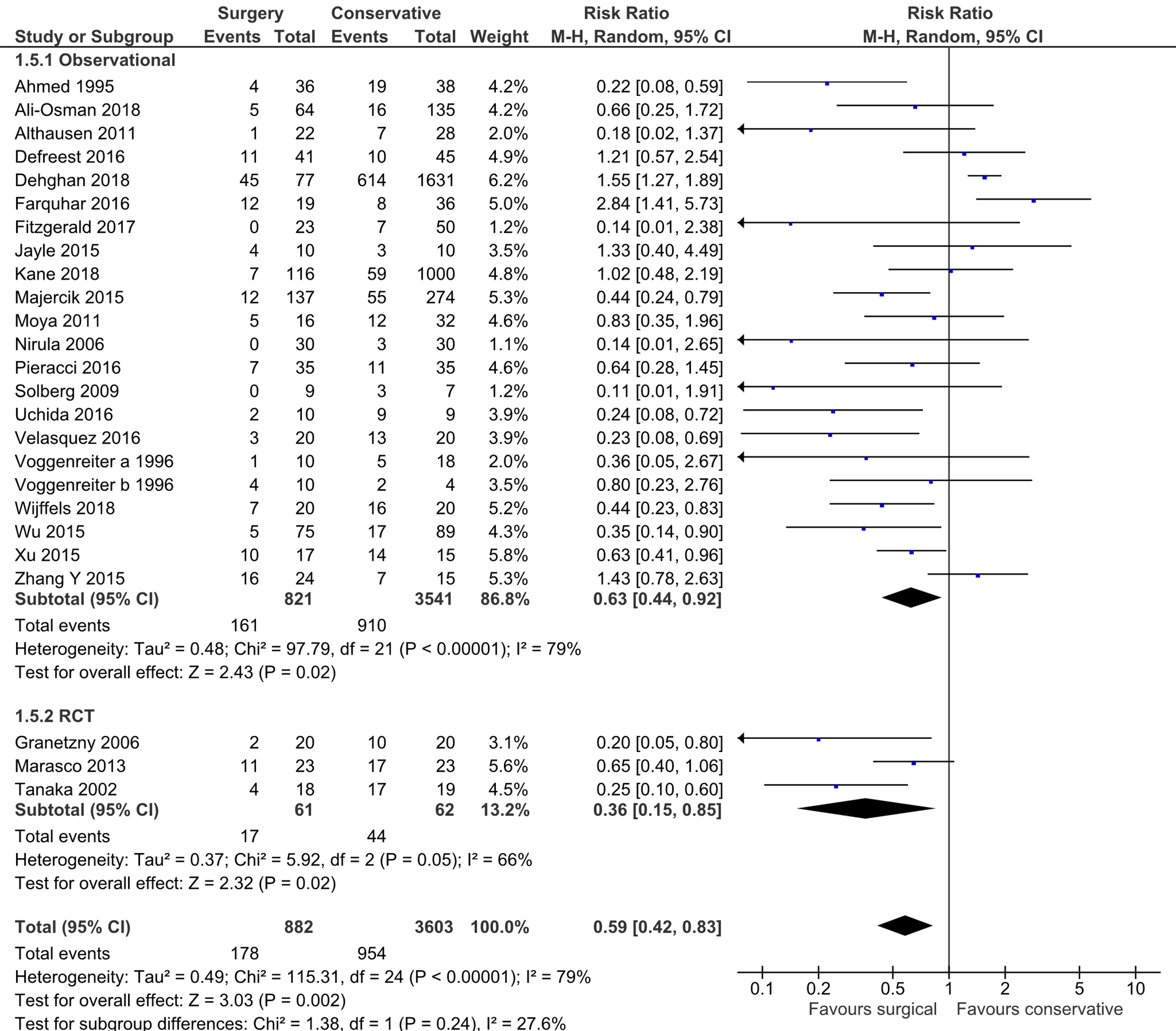

Supplement: Supplementary file 1 — Supplementary material 1 (DOCX 9071 KB) [file 68_2018_1020_MOESM1_ESM.docx]
